# A cross-sectional examination of socio-demographic and school-level correlates of children’s school travel mode in Ottawa, Canada

**DOI:** 10.1186/1471-2458-14-497

**Published:** 2014-05-23

**Authors:** Richard Larouche, Jean-Philippe Chaput, Geneviève Leduc, Charles Boyer, Priscilla Bélanger, Allana G LeBlanc, Michael M Borghese, Mark S Tremblay

**Affiliations:** 1 Healthy Active Living and Obesity Research Group, Children’s Hospital of Eastern Ontario Research Institute, 401 Smyth Road, Room R242, Ottawa, ON K1H 8 L1, Canada; 2 Department of Pediatrics, University of Ottawa, Ottawa, Canada

**Keywords:** Active travel, Safe routes to school, Social ecological models, School policies, Built environment

## Abstract

**Background:**

Active school transport (AST) is an important source of children’s daily physical activity (PA). However, decreasing rates of AST have been reported in multiple countries during the last decades. The purpose of the present study was to examine the socio-demographic and school-level correlates of AST.

**Methods:**

A stratified sample of children (N = 567, mean age = 10.0 years; 57.8% female) was recruited in the Ottawa area. Four sources of data were used for analyses: 1) child questionnaire including questions on school travel mode and time; 2) parent questionnaire providing information on household socio-demographic characteristics; 3) school administrator survey assessing school policies and practices pertaining to PA; and 4) school site audit performed by the study team. Generalized linear mixed models were used to identify socio-demographic and school-level correlates of AST while controlling for school clustering.

**Results:**

Individual factors associated with higher odds of AST were male gender (OR = 1.99; 95% CI = 1.30-3.03), journey time <5 minutes vs. >15 minutes (OR = 2.26; 95% CI = 1.17-4.37), and 5–15 minutes vs. >15 minutes (OR = 2.27; 95% CI = 1.27-4.03). Children were more likely to engage in AST if school administrators reported that crossing guards were employed (OR = 2.29; 95% CI = 1.22-4.30), or if they expressed major or moderate concerns about crime in the school neighbourhood (OR = 3.34; 95% CI = 1.34-8.32). In schools that identified safe routes to school and where traffic calming measures were observed, children were much more likely to engage in AST compared to schools without these features (OR = 7.87; 95% CI = 2.85-21.76). Moreover, if only one of these features was present, this was not associated with an increased likelihood of AST.

**Conclusion:**

These findings suggest that providing crossing guards may facilitate AST. Additionally, there was a synergy between the identification of safe routes to school and the presence of traffic calming measures, suggesting that these strategies should be used in combination.

## Background

Previous systematic reviews indicate that children and youth using active modes of transportation such as walking or cycling to travel to/from school (e.g., active school transport; AST) accumulate more daily physical activity (PA) than those who are driven by car or bus [1,2]. In addition, children who cycle to/from school have higher cardiovascular fitness than their peers using motorized travel modes [1,3,4]. Other investigators have also reported that AST was associated with greater academic achievement [5] and reduced stress [6].

Together, these findings suggest that AST should be promoted as a strategy to improve children’s health and well-being. However, the proportion of children engaging in AST has decreased markedly over the last few decades in many countries including Australia [7], Canada [8], Switzerland [9], and the United States [10]. Furthermore, interventions aimed at promoting AST have generally achieved only modest shifts from car travel to AST [11,12].

To inform the development of more effective interventions, a better understanding of the correlates of AST is warranted. Current theoretical models that seek to predict AST are typically based on the social ecological approach [13,14] which posits that behaviour is determined by the interactions between multiple levels of influence. These include characteristics of the individual, the social environment, the built environment, public policies, and the physical environment [15,16]. To date, few studies have assessed school-level factors associated with AST while controlling for relevant individual characteristics and clustering within schools [17-19]. Rooted in a social ecological approach, such investigations have the potential to unravel interactions between multiple levels of influence and to identify promising intervention strategies [20].

Therefore, the present study investigated the socio-demographic and school-level correlates of AST among 10-year-old children in Ottawa, Canada. Data were collected from multiple sources (child and parent questionnaire, school administrator survey and school audit) in order to examine multiple levels of influence. The overarching goal was to identify factors accounting for the between-school variation in AST to inform the development of future interventions.

## Methods

### Participants

The present study used data from the Canadian site of the International Study of Childhood Obesity, Lifestyle and the Environment (ISCOLE). Based on the social-ecological model, this 12-country cross-sectional study aimed to collect data on the correlates of childhood obesity at the individual, family, neighbourhood, and school environment levels. Greater details concerning the study design are available elsewhere [21]. The Canadian site consisted of 567 grade five students (239 boys and 328 girls; 9–11 years of age) recruited between September 2012 and May 2013 in 26 schools within the Ottawa region. The Ottawa-Gatineau region is the fourth largest census metropolitan area in Canada, and the population is predominantly English speaking, albeit with a large French speaking minority [22]. Schools were stratified into four groups: English Public (n = 393; 69.3%), French Public (n = 60; 10.6%), English Catholic (n = 75; 13.2%), and French Catholic (n = 39, 6.8%). The response rate was 50%. This study was approved by the research ethics boards of the Children’s Hospital of Eastern Ontario, and the participating school boards. Written informed parental consent and child assent were obtained for all participants.

### Procedures

First, trained study staff administered a child questionnaire in schools [21]. Travel mode was assessed with one item (“*in the last week you were in school, the MAIN part of your journey to school was by*”). Response options were: 1) walking; 2) bicycle, rollerblade, skateboard, scooter; 3) bus, train, tram, underground, or boat; 4) car, motorcycle, or moped; 5) other. Children who reported “other” were asked to specify their travel mode. Children were also asked to report their usual school journey time. Categories were: 1) <5 minutes; 2) 5–15 minutes; 3) 16–30 minutes; 4) 31 minutes to 1 hour; 5) >1 hour.

Second, socio-demographic variables were obtained through a parent questionnaire [21] (Table 1). Information on annual household income (eight levels), mother’s education (six levels), number of functioning motorized vehicles (five levels), and the child’s gender and ethnicity were examined in the present analyses. Two school-level socio-demographic variables were considered in analyses: school language (English or French) and school board (Public or Catholic).

**Table 1:** Socio-demographic correlates of active school transport

**Variable**	**Categories**	**Frequency**	**Percentage**	**OR**	**95% CI**	**p**
Gender	Boys	239	42.2	1.98	1.31-2.98	.001
	Girls	328	57.8	Reference		
School travel mode	Active	199	35.1	N/A		
	Public transport	368	64.9			
School travel time	<5 minutes	134	23.6	2.39	1.24-4.63	.010
	5-15 minutes	289	51.0	2.44	1.37-4.34	.002
	>15 minutes	144	25.4	Reference		
Household income	<$60,000	105	19.3	0.77	0.40-1.49	.439
	$60,000-$139,999	231	42.4	0.87	0.54-1.40	.552
	≥$140,000	209	38.3	Reference		
Mother’s education	<College	85	15.2	1.52	0.84-2.76	.169
	College	142	25.4	0.72	0.43-1.21	.216
	University	331	59.3	Reference		
Motorized vehicles	≤1	236	42.1	1.25	0.81-1.94	.321
	≥2	325	57.9	Reference		
School language	French	99	17.5	0.19	0.06-0.64	.007
	English	468	82.5	Reference		
School board	Catholic	114	20.1	0.61	0.09-1.06	.061
	Public	453	79.9	Reference		

Note: Odds ratios for engaging in active transportation were estimated using generalized linear mixed models adjusted only for school clustering.

Third, the school environment was assessed with a questionnaire completed by a school administrator (e.g., the principal). Questionnaire items were taken from the School Health Environment Survey which is a component of the Canadian School Health Action, Planning and Evaluation System [23]. Satisfactory test-retest reliability and convergent validity between school administrators and teachers was noted in a separate purposive sample of Ontario educators, as reported in details elsewhere [24]. Of particular interest, administrators were questioned about the existence of school policies related to PA and healthy eating, on school facilities that may influence children’s mobility (i.e., the presence and safety of bike racks) and on school-based promotion of AST (i.e., identification of safe routes to school, organization of events such as walk to school days). They were also asked whether they perceived religious tensions, litter in the streets, drugs/drinking, gangs, heavy traffic, vacant/shabby houses, and crime as a problem in the school neighbourhood on 4-point Likert scales ranging from “major problem” to “not a problem”, with a “don’t know” option for each question. Eighteen variables from the administrator questionnaire were considered for the present analyses (Table 2).

**Table 2:** School-level correlates of active school transport, as reported by school administrators

**Variable**	**Categories**	**Frequency**	**Percentage**	**OR**	**95% CI**	**p**
Physical activity policies*	Existing policies	501	88.4	1.99	0.38-10.46	.415
	Practices	66	11.6	Reference		
Healthy eating policies*	Existing policies	499	88.0	0.51	0.10-2.58	.413
	Practices	68	12.0	Reference		
Identify safe routes to school	Yes	289	51.0	3.63	1.39-9.44	.008
	No	278	49.0	Reference		
Provides crossing guards	Yes	284	50.1	5.75	2.52-13.10	<.001
	No	283	49.9	Reference		
Designate car free zone	Yes	238	42.0	1.52	0.52-4.49	.448
	No	329	58.0	Reference		
Allow students to bring bicycles*	Yes	534	94.2	3.29	0.48-22.42	.224
	No	33	5.8	Reference		
Allow students to bring small wheel vehicles^†^	Yes	351	61.9	2.72	0.94-7.84	.064
	No	216	38.1	Reference		
Encourage use of helmets and safety gear*	Yes	544	95.9	1.17	0.14-9.53	.887
	No	23	4.1	Reference		
Organize events (i.e. walk to school days)	Yes	131	23.1	1.25	0.38-4.13	.718
	No/don’t know	436	76.9	Reference		
Access to bike racks during school hours*	Yes, on grounds only	527	92.9	2.98	0.50-17.70	.229
	No	40	7.1	Reference		
Bikes stored in a secure area	Yes	383	67.5	1.38	0.45-4.22	.571
	No/don’t know	184	32.5	Reference		
Religious tension perceived as problem*	Major/moderate	43	7.6	1.73	0.29-10.13	.544
	Minor	153	27.0	1.68	0.43-6.62	.459
	No problem or don’t know	372	65.4	Reference		
Garbage/litter perceived as problem*	Major/moderate	52	9.2	3.31	0.66-16.52	.145
	Minor	235	41.4	1.69	0.52-5.47	.380
	No problem or don’t know	280	49.4	Reference		
Drugs/drinking perceived as problem*	Major/moderate	30	5.3	4.36	0.78-24.54	.094
	Minor	92	16.2	0.72	0.14-3.56	.683
	No/don’t know	445	78.5	Reference		
Gangs perceived as problem*	Major/moderate	25	4.4	3.98	0.64-24.87	.140
	Minor	45	7.9	1.47	0.27-7.99	.654
	No problem or don’t know	497	87.7	Reference		
Traffic perceived as problem	Major/moderate	126	22.2	3.41	0.86-13.56	.081
	Minor	254	44.8	1.30	0.39-4.37	.671
	No problem or don’t know	187	33.0	Reference		
Vacant/shabby housing perceived as problem*	Major/moderate	28	4.9	4.11	0.73-23.32	.110
	Minor	43	7.6	4.26	0.58-31.15	.153
	No problem or don’t know	496	87.5	Reference		
Crime perceived as problem*	Major/moderate	33	5.8	5.99	1.24-28.87	.026
	Minor	190	33.5	1.89	0.57-6.29	.302
	No problem or don’t know	344	60.7	Reference		

Note: Odds ratios for engaging in active transportation were estimated using generalized linear mixed models adjusted only for school clustering. *Denotes that fewer than 5 schools have one of the categories being compared; these results should be interpreted with caution. ^†^Includes skateboard, rollerblades, scooters.

Fourth, a school audit was performed by a single trained examiner at each participating school [21] to examine opportunities for PA in the school environment. A photo dictionary was created to standardize data collection procedures. Most of the included items were taken from the Sport, Physical Activity and Eating Behaviour: Environmental Determinants in Young People (SPEEDY) study audit [25] which has shown acceptable inter-rater reliability and construct validity (as assessed with accelerometry-measured PA). Fourteen items were retained for the present analyses (Table 3). Twelve items pertained to walking and cycling provisions in the school environment. The auditor was asked to indicate whether these items were visible from any school entrance. A similar item on the visibility of fast food restaurants from any of the school entrances was added by the ISCOLE team [21]. Finally, land use (as perceived by the auditor) was assessed with a single question (“*Is the area around the school predominantly*?”): 1) “residential”; 2) “open fields/commons/parks”; 3) “business/retail”; 4) “a mixture of different land uses”. Previous research shows that land use mix is an important correlate of adults’ active transport, although its influence on children’s mobility remains equivocal [26].

**Table 3:** **School-level correlates of active school transport, as observed in the school audit**^†^

**Variable**	**Categories**	**Frequency**	**Percentage**	**OR**	**95% CI**	**p**
Predominant land use around school*	Residential	508	89.6	0.64	0.14-2.95	.643
	Others	59	10.4	Reference		
Place where parents can stop and drop children off*	No	8	1.4	7.69	0.72-81.90	.091
	Yes	559	98.6	Reference		
Place where parents can park their cars*	No	88	15.5	2.32	0.44-12.16	.319
	Yes	479	84.5	Reference		
Bus stop	No	85	15.0	2.00	0.57-7.02	.276
	Yes	482	85.0	Reference		
Cycle lane separated from the road*	No	567	100.0	N/A		
	Yes	0	0.0			
Cycle lane on the road	No	531	93.7	0.46	0.03-6.48	.556
	Yes	36	6.3	Reference		
Sidewalks on both sides^‡^	No	93	16.4	3.07	0.85-11.04	.086
	Yes	474	83.6	Reference		
Sidewalks on one side only	No	328	57.8	0.37	0.13-1.04	.060
	Yes	239	42.2	Reference		
Marked pedestrian crossing	No	104	18.3	3.17	0.98-10.27	.054
	Yes	463	81.7	Reference		
Traffic calming measures	No	333	58.7	4.05	1.52-10.79	.005
	Yes	234	41.3	Reference		
School warning sign for road users*	No	11	1.9	1.12	0.07-19.16	.937
	Yes	556	98.1	Reference		
Road safety sign	No	56	9.9	1.29	0.32-5.12	.722
	Yes	501	90.1	Reference		
Route sign for cyclists	No	437	77.1	0.55	0.16-1.91	.345
	Yes	130	22.9	Reference		
Fast food restaurants	No	352	62.1	0.41	0.14-1.20	.102
	Yes	215	37.9	Reference		

Note: Odds ratios for engaging in active transportation were estimated using generalized linear mixed models adjusted only for school clustering. *Denotes that fewer than 5 schools have one of the categories being compared; these results should be interpreted with caution. ^†^For all school audit items except land uses, the auditor was asked to specify if any of the items were visible from any school entrance. ^‡^If any of the road segments observed had sidewalks on both sides, this item was coded as yes.

### Data treatment

Children’s travel mode was dichotomized as active (walk, cycle, etc.) vs. inactive (car, bus, etc.). Participants who reported ‘other’ modes (n = 2) indicated running or jogging; hence, they were classified as active travelers. Given the large number of categories in the questionnaires, socio-demographic variables were recoded based on the observed distributions (Table 1). School travel time was categorized as 1) <5 minutes; 2) 5–15 minutes; 3) >15 minutes. Most journeys longer than 15 minutes were done by car or bus, so collapsing the response options did not have a marked influence on the results. Annual household income was categorized as 1) < $60,000; 2) $60,000-$139,999; 3) ≥ $140,000. Mother’s education was categorized as 1) < college; 2) college; and 3) university. Motorized vehicle ownership was dichotomized as one or none versus two or more. Given that few administrators perceived major safety problems in the school neighbourhood, their responses to these seven items were recoded as 1) major/moderate problem; 2) minor problem; and 3) not a problem/don’t know. In addition, if administrators did not know whether their school identified safe routes to school, it was assumed that they did not. This assumption was based on the fact that school travel planning [27] is a whole-of-school intervention that mobilises school administrators, teachers, students, and the broader community; thus, administrators would be expected to know if such a scheme was in place at their school. Finally, land use was recoded as “residential” versus “others”.

### Statistical analyses

Generalized linear mixed models (GLMM) with a binomial distribution and logit link were used to examine the socio-demographic and school-level correlates of children’s travel mode (e.g., active vs. inactive) following analytical procedures described by Cerin [20]. First, a model was fitted with only school entered as a random effect to determine the within-school intra-class correlation coefficient (ICC). Specifically, the greater the ICC, the more students attending the same school used the same travel mode. Second, socio-demographic variables were added as fixed effects in the model adjusted for school clustering. Only variables that were significant at the 0.05 level were kept in the model, and interactions among these variables were examined. Third, school-level factors were individually added to the model developed in step 2. Only school-level factors that were significant at the 0.05 level were retained for the final model. The school audit item pertaining to bike lanes separated from the road was omitted, because this feature was absent in all schools. Fourth, a final model was fitted with the socio-demographic and school level correlates that were found to be significantly associated with AST, and interactions among these variables were examined. All analyses were performed using IBM SPSS version 21 (Armonk, United States).

## Results

Descriptive characteristics of the sample are shown in Tables 1, 2, 3. 35.1% of participants reported that they regularly engaged in AST (33.9% walking and 1.3% other active modes). Conversely, 38.6% of participants reported using public transportation (i.e., school buses) and 26.3% traveled by car. Approximately half of the participants reported school travel times between 5 and 15 minutes while the remainder was rather evenly distributed across the <5 minutes and ≥15 minutes categories. Parents reported relatively high income and over half of the mothers had received university education. 42.1% of parents owned ≤1 motorized vehicles while 57.9% owned ≥2 vehicles. In general, most school administrators indicated having written PA and healthy eating policies in place, and their school reportedly encouraged AST through various strategies. School administrators tended to perceive the school neighbourhood as relatively safe, and the most frequently reported concern was traffic safety. Broadly speaking, the school audit revealed that most schools were located in predominantly residential areas and there was generally good infrastructure to support walking. However, provision of cycling infrastructure was very poor with few bike lanes and traffic signs for cyclists.

The initial GLMM indicated large clustering of AST at the school level (ICC = 0.31; *p* = 0.005), emphasizing the importance of controlling for school clustering. In models adjusted only for school clustering, boys (OR = 1.98; 95% CI = 1.31-2.98) and participants reporting school trip durations of <5 minutes (OR = 2.39; 95% CI = 1.24-4.63) or 5–15 minutes (OR = 2.44; 95% CI = 1.37-4.34) were significantly more likely to engage in AST compared to girls and those with travel times >15 minutes (Table 1). No interactions were observed between gender and school travel time (data not shown). Income, mother’s education, car ownership and school board (e.g., public or catholic) were not associated with AST. However, lower odds of AST were noted in children attending French schools (OR = 0.19; 95% CI = 0.06-0.64).

Table 2 shows that three of the administrator survey items were significantly associated with AST. Specifically, children were more likely to engage in AST if administrators reported that the school identified safe routes to school (OR = 3.63; 95% CI = 1.39-9.44) or that crossing guards were employed (OR = 5.75; 95% CI = 2.52-13.10). If administrators perceived that crime was a major or moderate problem in the school neighbourhood children were more likely to engage in AST than if crime was not perceived as a problem (OR = 5.99; 95% CI = 1.24-28.87). This effect should be interpreted cautiously given that only three administrators (representing 33 students) perceived crime as a major or moderate problem. Finally, if traffic calming measures (i.e., speed bumps, narrower lanes) were observed during the school audit, children were significantly more likely to be active travelers (OR = 4.05; 95% CI = 1.52-10.79).

**Figure 1:**
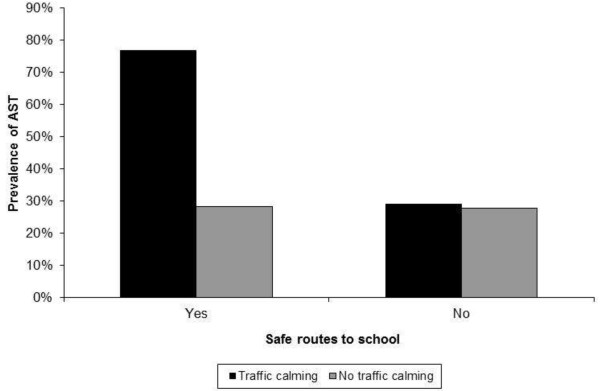
**Joint association of safe routes to school and traffic calming measures with active school transport.** Note: School administrators were asked whether their school identified safe routes for children to travel. The presence of traffic calming measures around the school was observed as part of a standardized audit performed by study staff. AST = Active school transport.

In the fully adjusted model (Table 4), the observed associations of gender and school travel time remained significant and their effect sizes remained virtually unchanged. Specifically, boys (OR = 1.99; 95% CI = 1.30-3.03) and participants reporting school trip durations of <5 minutes (OR = 2.26; 95% CI = 1.17-4.37) or 5–15 minutes (OR = 2.27; 95% CI = 1.27-4.03) were more likely to engage in AST. The provision of crossing guards was also associated with greater odds of AST (OR = 2.29; 95% CI = 1.22-4.30). Again, if administrators perceived that crime was a major or moderate problem in the school neighbourhood, children were more likely to use AST (OR = 3.34; 95% CI = 1.34-8.32). Finally, an interaction was observed between the identification of safe routes to school and the presence of traffic calming measures (Figure 1). If both features were present (i.e., safe routes to school combined with traffic calming measures), children were almost eight times more likely to engage in AST (OR = 7.87; 95% CI = 2.85-21.76). If only one of these features were present, it was not associated with a greater likelihood of AST. Differences between children attending French vs. English schools were no longer significant. The final model explained most of the clustering of AST at the school level as shown by the very small ICC of 0.01 (p = 0.718).

**Table 4:** Multivariate associations of socio-demographic and school-level factors with children’s engagement in active transportation

**Variable**	**Categories**	**OR**	**95% CI**	**p**
Gender	Boys	1.99	1.30-3.03	.002
	Girls	Reference		
School travel time	<5 minutes	2.26	1.17-4.37	.015
	5-15 minutes	2.27	1.27-4.03	.006
	>15 minutes	Reference		
School language	French	0.71	0.31-1.62	.411
	English	Reference		
**School administrator survey variables**				
Identify safe routes to school	Yes	1.03	0.52-2.04	.935
	No	Reference		
Provides crossing guards	Yes	2.29	1.22-4.30	.010
	No	Reference		
Crime perceived as problem*	Major/moderate	3.34	1.34-8.32	.010
	Minor	1.19	0.68-2.08	.543
	Not a problem	Reference		
**School audit variables**				
Traffic calming measures	Yes	1.06	0.52-2.16	.865
	No	Reference		
Safe routes to school X Traffic calming interaction	Both yes	7.87	2.85-21.76	<.001
	Both no	Reference		

Note: Odds ratios for engaging in active transportation were estimated using generalized linear mixed models adjusted only for school clustering. Only variables that were significantly associated with active school transport in the bivariate models (e.g., Tables 1, 2, 3) were included in the multivariate model. In the empty model, the school ICC was 0.31 (p < 0.001) while in the fully adjusted model, it was 0.01 (p = 0.718). *Denotes that fewer than 5 schools have one of the categories being compared; these results should be interpreted with caution.

## Discussion

The present study assessed the socio-demographic and school-level correlates of AST among 10-year-old children in Ottawa, Canada. Analyses indicated that approximately 31% of the variance in AST was explained at the school level. The final model shows that boys, children whose school journey was shorter, and those attending schools where crossing guards were employed were approximately twice as likely to engage in AST. When school administrators reported that crime in the school neighbourhood was a major or moderate problem, children were over three times more likely to engage in AST. Of particular interest, there was a synergy between the identification of safe routes to school (as reported by administrators) and the presence of traffic calming measures (ascertained by the study team). Specifically, when both of these characteristics were present, children were almost eight times more likely to engage in AST. Collectively, these findings should be relevant for policy-makers, education leaders, public health workers, and urban/transport planners interested in facilitating children’s AST.

To our knowledge, no previous study has reported such a synergy between the identification of safe routes to school and the presence of traffic calming measures in the vicinity of the school. Nevertheless, this finding is consistent with a recent evaluation of the Safe Routes to School program in Oregon, United States [28]. Specifically, McDonald and colleagues [28] reported that more comprehensive interventions (including education and encouragement programs combined with infrastructure improvements) were more effective, achieving 5–20 percentage points increases in walking and cycling. Together, these findings suggest substantial interactions between the social environment and the built environment, as postulated by social ecological models [15,16]. Nevertheless, this relationship could also be driven by unmeasured variables such as the average distance between home and school [26,29] or neighbourhood walkability [17,30] which are known to influence travel behaviours. This underscores a need for further studies.

Interestingly, neither the identification of safe routes to school nor the presence of traffic calming measures was independently associated with children’s AST in the adjusted model. This finding may suggest that interventions focusing only on the promotion of AST or on infrastructure changes may be insufficient to trigger behaviour change. Of particular interest, the Canadian school travel planning model uses a comprehensive approach where school specific interventions are developed to change travel behaviours and improve safety based on input from members of the school community (e.g., students, parents, and teachers) and other stakeholders [26]. Recent evaluations of this approach have revealed either a modest increase in AST [26] or no significant differences in travel behaviours [12]. These somewhat disappointing results may be attributable to the fact that schools were followed for only one year, and this time frame is likely too short for comprehensive school travel plans to be fully implemented, or their impact realized [12]. This hypothesis is supported by the results of the New Zealand school travel planning intervention which indicated significant increases in AST after three years of implementation, but not after only one year [31]. Hence, there remains a need for longer investigations examining the effectiveness of this program as well as moderators and mediators of behaviour change.

AST was also strongly associated with the presence of crossing guards, as reported by the school administrators. This finding is consistent with recent studies in the city of Toronto [32], the United States [33], and the United Kingdom [19]. Interestingly, hiring crossing guards may represent an easier (and relatively inexpensive) strategy to encourage AST compared to major built environment changes [32].

Notably, children were more likely to engage in AST if school principals perceived that crime was a major or moderate problem in the school neighbourhood. While counter-intuitive, this finding is in agreement with previous research reporting a greater likelihood of AST if parents did not perceive their neighbourhood as safe [34] or as an excellent area to raise a child [35]. Other researchers also reported higher rates of AST in areas characterized by greater incivilities [36]. Nevertheless, longitudinal studies are warranted to examine whether such associations may be attributable to reverse causality; e.g., school officials may be more concerned about neighbourhood safety if they are aware that a large proportion of students walk or cycle to/from school. In addition, several North American studies have reported higher rates of AST in low SES areas [18,37,38] where motorized travel may not be an available option. In the present study, area deprivation (as estimated using the median household income of the census tract in which the schools were located) was not associated with AST (data not shown).

The observation that children who had longer journeys to school were less likely to engage in AST is consistent with previous literature reviews indicating that long distance between home and school is a major barrier to AST [26,29]. Furthermore, Torres et al. [39] reported lower rates of AST among children attending English schools in Montréal and Trois-Rivières (Québec) and noted that these children traveled greater distances to/from school. Our results show a similar association whereby children attending minority language schools (in this case, French) were approximately five times less likely to engage in AST after adjustment for school clustering. This association was no longer significant in the fully-adjusted model which included school travel time as a proxy for home-school distance. While it may not be feasible for children living far away from their school to do the entire trip on foot, a drop off zone could be designated within a “walkable” distance, so that children who are driven to school by car or bus can engage in some AST [40]. For example, a partnership was successfully developed to allow school buses to use the parking lot of a nearby church as part of a Safe Routes to School intervention in Atlanta, United States [41].

In the present study, boys were twice as likely as girls to engage in AST. This observation is consistent with many North American studies reporting higher rates of AST in boys [18,37,42,43]. Furthermore, at any given age, boys generally have greater independent mobility than girls [44,45]. Independent mobility refers to “the freedom of children to travel around their own neighbourhood without adult supervision” [46] and it has been repeatedly shown to be associated with greater AST and PA levels [47-49]. However, in Denmark, where cycling to school is much more prevalent and safer, no gender gaps have been reported [3].

The present findings should be interpreted cautiously given the cross-sectional study design which makes it impossible to determine the direction of the observed relationships. Moreover, the correlates of children’s current travel mode may differ from those of travel behaviour change, emphasizing a need for longitudinal investigations. A second limitation to consider is that all participating schools were recruited in the Ottawa region, so it is unclear whether similar associations would have been found elsewhere. However, multiple studies of correlates of AST have found associations with variables such as distance [26,29] and the presence of crossing guards [19,32,33] in various jurisdictions. Third, although walking and cycling may have different correlates, it was unfeasible to examine them separately owing to the scarcity of cycling in the sample. Fourth, only environmental characteristics around the school were examined. Previous research suggests that characteristics of the home neighbourhood and the route between home and school may also influence children’s travel behaviours [13,19]. Fifth, the reliability and validity of the travel mode questions was not assessed. However, previous research suggests that similar questions have high test-retest reliability and convergent validity between child and parent reports [50,51]. Furthermore, three studies have examined the test-retest reliability of school travel time questions among children of this age, and they all reported high coefficients (ICC ranging from 0.70 to 0.94), indicating that children can provide reliable estimates [52-54]. Finally, the relatively small number of clusters (e.g., 26 schools) may have led to lesser precision in the estimates as suggested by the large confidence intervals observed for many of the school-level factors.

However, the examination of the relative influence of socio-demographic and school-level correlates of AST while controlling for school clustering is an important strength of the study. Previous Canadian studies had shown large between-school variation in AST [18,55,56]. By identifying the factors accounting for the clustering of AST at the school level, the present study provides valuable insights for future school-based interventions and policies. Second, school-level factors were examined both from the perspective of school administrators and from a school audit performed by the study team, thus providing complementary information on a wide range of potential correlates of AST. Third, the sample was stratified according to school language and school board. After adjustment for school clustering, children attending French schools were about five times less likely to engage in AST, suggesting that interventions and policies targeting this minority population may be needed. Finally, complete data were available for all of the variables included in the final model.

## Conclusion

This study found that boys, children reporting shorter school journeys, and those attending schools where crossing guards were employed or where school administrators expressed concerns about crime in the school neighbourhood were more likely to engage in AST. Furthermore, there was a strong synergy between the identification of safe routes to school and the presence of traffic calming measures in the school neighbourhood, suggesting that these strategies should be used in combination. Together, these variables explained the majority of the clustering of AST at the school level. Future longitudinal studies should examine these variables as potential correlates of travel behaviour change in the context of AST interventions.

## Competing interests

The authors declare that they have no competing interests.

## Authors’ contributions

RL, JPC and MST conceived the analysis plan. RL conducted the statistical analyses and drafted the manuscript. GL coordinated the study and CB, PB, AGL and MMB were involved in data collection. JPC and MST were the Principal Investigators for the Canadian ISCOLE site. All authors carefully reviewed and commented the first draft and approved the submitted version.

## Pre-publication history

The pre-publication history for this paper can be accessed here:

http://www.biomedcentral.com/1471-2458/14/497/prepub
